# Characterization of differential transcript abundance through time during *Nematostella vectensis* development

**DOI:** 10.1186/1471-2164-14-266

**Published:** 2013-04-19

**Authors:** Rebecca Rae Helm, Stefan Siebert, Sarah Tulin, Joel Smith, Casey William Dunn

**Affiliations:** 1 Ecology and Evolutionary Biology, Brown University, 80 Waterman Street, Box G-W, Providence, RI 02912, USA; 2 Eugene Bell Center for Regenerative Biology and Tissue Engineering, Marine Biological Laboratory, 7 MBL St., Woods Hole, MA 02543, USA; 3 Department of Molecular Biology, Cell Biology, and Biochemistry, Brown University, 185 Meeting Street, Providence, RI 02906, USA

**Keywords:** *Nematostella vectensis*, Transcriptome, Gene expression, Maternal to zygotic transition, Development

## Abstract

**Background:**

*Nematostella vectensis,* a burrowing sea anemone, has become a popular species for the study of cnidarian development. In previous studies, the expression of a variety of genes has been characterized during *N. vectensis* development with *in situ* mRNA hybridization. This has provided detailed spatial resolution and a qualitative perspective on changes in expression. However, little is known about broad transcriptome-level patterns of gene expression through time. Here we examine the expression of *N. vectensis* genes through the course of development with quantitative RNA-seq. We provide an overview of changes in the transcriptome through development, and examine the maternal to zygotic transition, which has been difficult to investigate with other tools.

**Results:**

We measured transcript abundance in *N. vectensis* with RNA-seq at six time points in development: zygote (2 hours post fertilization (HPF)), early blastula (7 HPF), mid-blastula (12 HPF), gastrula (24 HPF), planula (5 days post fertilization (DPF)) and young polyp (10 DPF). The major wave of zygotic expression appears between 7–12 HPF, though some changes occur between 2–7 HPF. The most dynamic changes in transcript abundance occur between the late blastula and early gastrula stages. More transcripts are upregulated between the gastrula and planula than downregulated, and a comparatively lower number of transcripts significantly change between planula and polyp. Within the maternal to zygotic transition, we identified a subset of maternal factors that decrease early in development, and likely play a role in suppressing zygotic gene expression. Among the first genes to be expressed zygotically are genes whose proteins may be involved in the degradation of maternal RNA.

**Conclusions:**

The approach presented here is highly complementary to prior studies on spatial patterns of gene expression, as it provides a quantitative perspective on a broad set of genes through time but lacks spatial resolution. In addition to addressing the problems identified above, our work provides an annotated matrix that other investigators can use to examine genes and developmental events that we do not examine in detail here.

## Background

*Nematostella vectensis* is a burrowing, estuarine sea anemone that has been an important model system for embryonic development in Cnidaria, and was the first cnidarian to have a draft genome sequence available [1]. Mature *N. vectensis* liberate gametes into the water. Cleavage begins roughly two hours after fertilization, with gastrulation occurring roughly 20 hours post fertilization (HPF) at 18°C [2]. Embryos develop into swimming planula larvae. After variable time in the water column (roughly 10 days post fertilization (DPF)), the planulae metamorphose and settle to the benthos as young polyps.

There have been extensive studies of gene expression throughout development in *N. vectensis*, based largely on *in situ* mRNA hybridization [e.g. [2-6]. These studies have provided detailed pictures of differential spatial expression, as well as qualitative assessments of changes in expression through time. In this paper, we complement these spatial expression studies with quantitative RNA-seq analyses of whole embryos through time. This approach provides no spatial resolution, but allows for a temporal analysis of changes in expression across the whole embryo for previously annotated genes [1]. High-throughput quantitative expression studies have been conducted in a handful of other cnidarian species. Previous quantitative sequencing work has focused on differential gene expression between different zootypes within a colony [7], the response of developing coral larvae to temperature stress [8], and the response to ocean acidification [9]. However, none of these studies focus on patterns in gene expression through multiple stages of development.

We sampled *N. vectensis* embryos at six time points through the course of development: 2 HPF, 7 HPF, 12 HPF, 24 HPF, 5 (DPF), and 10 DPF. The interval from 2–7 HPF captures early cleavage events through approximately 128 cells. 7–12 HPF encompases prawn chip stages I-V [2]. 12–24 HPF includes the onset of gastrulation. The 24 HPF - 5 DPF interval spans development from a gastrula to a planula. In the interval from 5–10 DPF the animals develop tentacle buds and settle.

Each of these six time points was sampled from two replicated spawning events, giving a total of twelve samples. Expression in each of the twelve samples was then quantified with RNA-seq. This project design allows us to characterize broad patterns in expression through time, as well as address specific questions about transcriptional dynamics over the course of development. We focus in particular on the maternal to zygotic transition, with a brief overview of gastrulation.

## Results and discussion

### Sequencing, mapping, and consistency across replicates

On average, 13.77 million sequence reads passed the Illumina chastity filter for each sample. We deposited these reads at the NCBI Sequence Read Archive (Project: PRJNA189768). Between 28-69% of these reads mapped to nuclear ribosomal sequences (18S or 28S) or the mitochondrial genome, and were not considered in the statistical analysis. Reads mapped to 23,044 of the 26,514 sequences in the reference (full edited reference: Additional file 1). Dispersion was low in the edgeR analyses (ranging from 0.008 to 0.016), indicating low variation between the replicates even though they were from two different clutches that were spawned months apart.

The count matrix, along with results of statistical analyses and other annotations, is available as Additional file 2. The R code for several example analyses of this matrix are presented in Additional file 3. These vignettes can be used as starting points for analyses beyond those presented here, such as more detailed investigations of particular time points, genes, or genes with gene ontology (GO) annotations of particular interest.

### Patterns in transcript abundance through time

**Figure 1:**
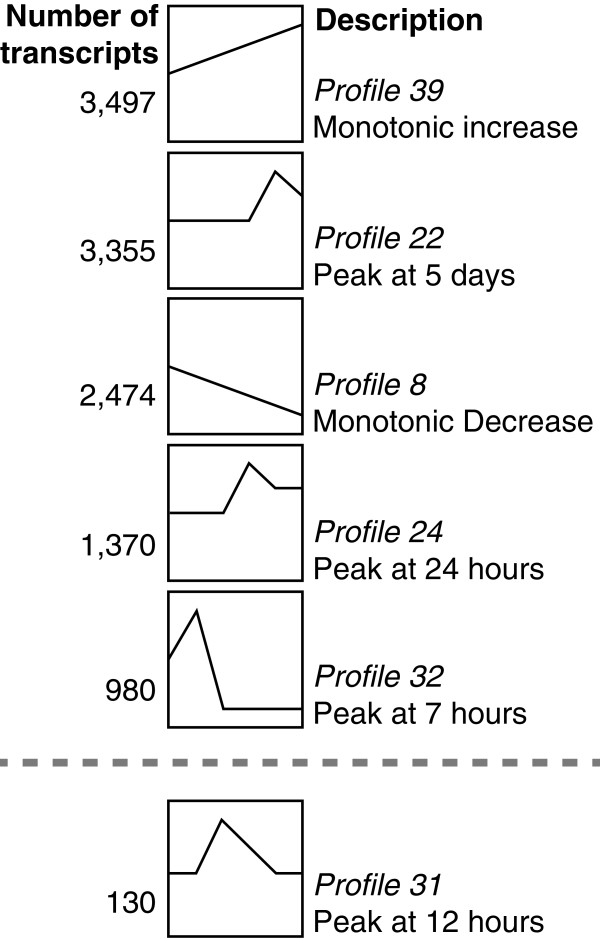
**Selected STEM profiles.** The five most abundant patterns of changes in transcript abundance through time, ranked by decreasing number of transcripts. Stem pattern 31, which is discussed in the text, is also shown below the dashed line. The full set of STEM profiles are shown in Additional file 4. The vertical axis is relative transcript abundance. The horizontal axis is developmental time, with the 6 time points arranged consecutively on the horizontal axis of each plot, from the first time point (2 HPF) on the left and the last (10 DPF) on the right.

To get a broad overview of changes in transcript abundance through time we performed Short Time-series Expression Miner (STEM) analysis, which categorizes each transcript according to temporal patterns of expression (Figure 1). 17,383 transcripts received a STEM profile.

Among the top five expression profiles, are patterns of both monotonic increase and decrease, as well as peaks at 3 of the 4 intermediate time points (Figure 1). The most represented pattern is an increase in abundance through development (3,497 genes, Figure 1: Profile 39), which includes transcripts involved in ribosomal function e.g. JGI transcript ID: 234893, ID: 235818, ID: 236265), *wnt*-like transcripts (e.g. ID: 106241, ID: 211618, ID: 228651, ID: 230011) several possible Wnt receptor *frizzled*-like transcripts (ID: 221521, ID: 133025), neurotransmitters and receptors (ID: 10746, ID: 247614) and transcripts with a possible relationship to muscle structure/function (eg. ID: 125819, ID: 202060, ID: 211472). A monotonic decrease in transcript abundance is also among the top five most abundant plots (Figure 1: Profile 8), and these transcripts are discussed in greater detail below (under *Maternal Transcript Degradation*). Transcripts within a STEM peak at or after gastrulation (3,355 genes, Profile 22; 1,370 genes, Profile 24) include those related to laminins, which are possibly involved in gastrula epithelialization (eg. ID: 187372; ID: 208267, ID: 214923), as well as light sensing rhodopsins and opsins (eg. ID:189274, ID: 197433, ID: 201968). The fifth most abundant profile includes transcripts that peak at 7 hours and decrease over time (980, Figure 1: Profile 32). This category includes cellular growth factors (such as a possible fibroblast growth factor, ID: 211797), a *kruppel*-like factor (ID: 39461), and a possible CCR4 NOT transcription complex member (ID: 122610), discussed in greater detail in the *Maternal to Zygotic Transition* section.

### Transcripts with significant changes in abundance through time

**Figure 2:**
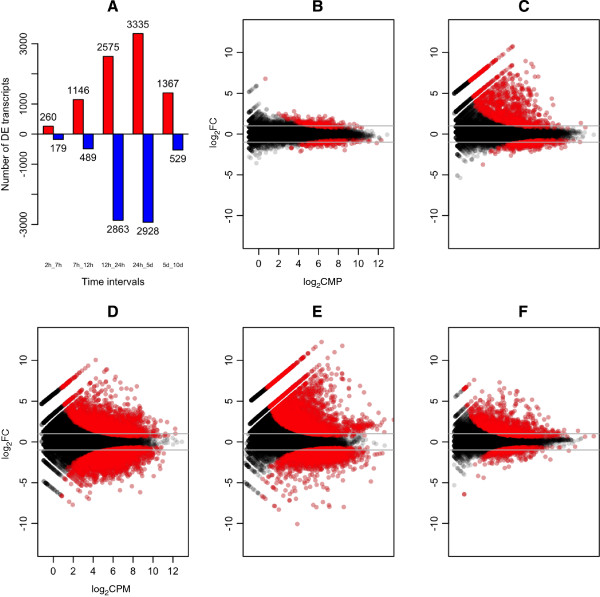
**Differential gene expression during early development of *N. vectensis*. A)** Number of transcripts that are significantly (p < 0.05) increasing (red), or decreasing (blue) through time within intervals. **(B-F)** Pairwise comparison Log_2_-fold-change vs log_2_CPM (counts per million) for the five pairwise comparisons between adjacent sampling times. The comparisons are between **(B)** 2 HPF and 7 HPF, **(C)** 7 HPF and 12 HPF, **(D)** 12 HPF and 24 HPF, **(E)** 24 HPF and 5 DPF and **(F)** 5 DPF and 10 DPF. Each point represents an individual transcript, red points indicate transcripts with significant (adjusted p-value < 0.05) differential expression. Positive log_2_-fold-change values indicate increased transcript abundance from the first to the second time point, negative log_2_-fold-change values indicate decreased transcript abundance from the first to second time point. Horizontal grey lines indicate 2-fold differences in expression.

9,456 of the 23,044 reference gene sequences with mapped reads (41.0%) were found to have differential gene expression (DGE; we only use this term when the difference is significant with an adjusted p-value < 0.05) across at least one of the time intervals examined here (Figure 2). There are relatively fewer genes with DGE between the first two time points (2–7 HPF), indicating that rates of zygotic transcription and selective mRNA degradation are low in this interval. Relative to this first interval, there are nearly four-fold more genes with DGE between 7–12 HPF. Most of these show an increase in transcript abundance, rather than a decrease. The number of genes with significant DGE is greater still in the interval between 12–24 HPF, with an almost equal number of genes with increased DGE (i.e., transcripts with significant increases in abundance) and decreased DGE (i.e., transcripts with significant decreases in abundance). The number of DGE genes continues to grow between 24 HPF - 5 DPF, with slightly more genes increasing in abundance than decreasing. Relative to the previous several intervals, a drop in the number of DGE genes is seen in the interval between 5 and 10 DPF, and is comparable to the number and proportion of DGE genes seen between 7–12 HPF.

### Gene ontology

In order to understand broad changes in functional categories of genes we performed a gene set enrichment analysis (Additional file 5). This analysis identified those GO categories that are overrepresented among genes with significant changes in expression over a given interval. The number of enriched GO categories in a given interval also lends some insight into the magnitude of change occurring in that timeframe.

Between 2–7 HPF 49 GO categories are significantly enriched. This is the smallest number of enriched categories of any interval, reflecting the relatively small number of significant transcriptional changes that occur between these time points. Many GO categories in this interval involve cell cycling; this is not surprising, as early development in many organisms is often characterized by rapid, maternally-run cell divisions [10]. Between 7–12 HPF 104 GO categories are enriched. These include categories related to translation, DNA binding (including transcription factor activity), and axis specification. These categories reflect the onset of zygotic transcription in this interval (see Table 1*,* and the *Timing of the Onset of Zygotic transcription* section below). The largest number of enriched GO categories is between 12–24 HPF, which comprises 339 categories. These include translation, intracellular processes (including endoplasmic reticulum targeting and protein localization), and processes related to membrane targeting and function. Between 24 HPF and 5 DPF, 312 categories are enriched, including categories related to transcription factor activity. Between 5–10 DPF a relative decrease in the number of enriched categories is observed, with only 87 categories. These include categories related to receptor activity, calcium binding, and muscle function, reflecting the development of neuronal and muscular features of the 10 day old polyps. Ribosomal activity is enriched in three of the intervals: 7–12 HPF, 12–24 HPF, and 24–5 DPF; this reflects the increasing translational needs of the embryo after the onset of zygotic transcription, until the interval between the planula and polyp stages, where these data imply the cellular ribosomal concentration reaches steady state.

**Table 1:** Select GO terms enriched between 7–12 HPF

**Rank**	**GO ID**	**Ontogeny term**	**Name**	**P-adjusted**	**Annotated**	**Decreasing**	**Increasing**
2	GO:0003735	Molecular function	structural constituent of ribosome	1.74E-37	175	0	77
7	GO:0006415	Biological process	translational termination	1.25E-25	54	0	36
8	GO:0006414	Biological process	translational elongation	9.01E-25	73	1	43
9	GO:0006614	Biological process	SRP-dependent cotranslational protein targeting to membrane	1.64E-24	54	0	37
30	GO:0006413	Biological process	translational initiation	3.63E-13	140	1	42
31	GO:0006412	Biological process	translation	6.57E-13	678	7	108
56	GO:0003700	Molecular function	sequence-specific DNA binding transcription factor activity	0.0003050592	587	8	56
61	GO:0003677	Molecular function	DNA binding	0.003018609	1429	40	86
72	GO:0009798	Biological process	axis specification	0.01298371	80	2	15
89	GO:0005667	Cellular component	transcription factor complex	0.02501985	860	19	67

GO enrichment of select transcripts that are changing in abundance during the major wave of zygotic gene expression. The GO-seq rank is given for each GO term, as well as the GO ID, and one of three possible GO ontology terms, which describe either the location, function or process the transcripts may be associated with. A description of the category is listed, the GO-seq p-adjusted value, the number of transcripts annotated within that category, and the relative number of transcripts whose abundance is decreasing or increasing through the interval.

### Maternal to zygotic transition

All sexually produced animals must pass control of the gene regulatory network from maternal factors deposited in the egg to newly synthesized gene products, synthesized after the embryo begins developing, of the zygotic genome. This transition is referred to as the Maternal to Zygotic Transition (MZT) and has been studied in depth in mammals, insects, fish, amphibians, nematodes, and echinoderms [10]. Research on the MZT in *Drosophila melanogaster* and the sea urchin *Strongylocentrotus purpuratus* has found that the transition is a combination of two phases: (1) the elimination of maternal RNAs and (2) the beginning of transcription from the zygotic genome [10]. Our data reveal key aspects of both processes in *N. vectensis*. First, we narrow the window of timing for embryonic transcription from the zygotic genome. Second, we identify transcripts that are present at the earliest time point and then decrease in abundance, and are likely the first maternal transcripts to be degraded. Other maternal transcripts may also be degraded, but the change hidden by concurrent embryonic transcription that masks the decline in maternal abundance. Third, we identify transcripts that increase in abundance between early time points, which are the first genes to be transcribed from the zygotic genome. Of these, we specifically focus on transcripts whose pattern of expression peaks early in development.

#### Timing of the onset of zygotic transcription

Only 260 transcripts show increasing abundance and DGE between 2–7 HPF, compared to 1,146 transcripts with increased DGE over the 7–12 HPF interval (Figure 2). These results suggest that the major onset of zygotic transcription begins between 7–12 HPF at 18°C. GO enrichment of the 7–12 hour time interval reflects this transition, and includes many categories related to early zygotic activity (Table 1).

#### Maternal transcript degradation

We took two approaches to identifying maternal transcripts that are degraded over time. First, we examined the transcripts with STEM profile 8, which have a monotonic decrease in abundance through time (Figure 1: Profile 8, Additional file 6; Additional file 4). Next, we identified those transcripts that decrease significantly between 2–7 HPF, regardless of changes in abundance over later intervals.

With STEM analysis we determined that 10.7% of mapped transcripts decrease over time (2,474 of 23,044), representing 15% of transcripts detected in the zygote (2,474 of 16,385). Of these 2,474 transcripts, 82% decreased significantly between at least two time points in development (2,053 of 2,474). Among them was a possible *N. vectensis* homologue of *mos2* (JGI *N. vectensis* transcript identification number (ID): 189257), which plays a role in oogenesis in the hydrozoan *Clytia hemisphaerica*[11]. Many transcripts associated with cell totipotency also decrease throughout development [12], including *vasa* domain containing transcripts (ID: 244465; ID: 230331), *piwi* (ID: 127599), and *tudor* (ID: 245679; ID: 224903; ID: 121235; ID: 7216).

After gaining general insights into broad patterns of transcripts with decreasing expression over the course of development, we next identified transcripts that decrease significantly between 2–7 HPF, regardless of subsequent changes. A set of 179 transcripts met this criterion (97 of these also decrease monotonically throughout the course of development).

Histone modification and rapid cell cycling are two proposed mechanisms by which expression of the zygotic genome is repressed in early development of bilaterians, and transcripts associated with these processes may be degraded preferentially in cnidarians as well [13]. Several chromatin remodeling homologues decrease significantly from 2–7 HPF. These include a putative histone modifier BRG1 associated factor (ID: 127783) that has been shown to be essential for the MZT in mice, [14]. Other putative histone modifiers include a histone methyltransferase (ID: 116282) and a possible member of a *male specific lethal*-like complex (ID: 113169) [15]. Cell cycle genes include a rootletin-like transcript (ID: 232574), which is associated with mitosis [16], and cyclin B-like transcript (ID: 208415). Maternally loaded RNA of Cyclin B is targeted for degradation in *Xenopus* embryos, possibly through an miRNA pathway [10]. The significant decrease in transcript abundance for these genes between 2–7 HPF suggests that maternal repression of zygotic expression in *N. vectensis* shares some conserved features with bilaterians, and that maternal repression is weakening in this interval.

#### Initiation of zygotic transcription

We next examined the initiation of zygotic transcription, first by identifying transcripts whose proteins may play a functional role in the degradation of maternal RNA, and second, by looking for transcripts that peak in abundance *only* at the 12 hour time point, and thus may be specific to the MZT or early embryonic development.

To isolate transcripts whose protein products may function in maternal RNA degradation, we examined transcripts that increase significantly either between 2–7 or 7–12 HPF. A *smaug* homolog (*smg*; ID: 240079) is present at low abundance in the zygote, and increases significantly at 12 HPF. In *D. melanogaster* Smg is a transcriptional regulator that binds to maternal transcripts, targeting them for degradation [17]. After *D. melanogaster* Smg binds to specific RNA sequences, it recruits the CCR4/POP2/NOT-deadenylase complex, which removes the poly(A) tail, thus signaling the RNA for degradation [10]. Two possible members of the CCR4/NOT transcription complex are significantly upregulated between 2–7 HPF (ID: 122631, ID: 104011) and a third between 7–12 HPF (ID: 195293). Which CCR4/NOT transcription complex Smg may be recruiting, if any, remains to be determined. However, these data suggest that the Smg CCR4/NOT transcription complex pathway for degradation of maternal RNAs may be present in cnidarians.

We next examined genes that appear to be expressed only at the 12 hour time point, and may be involved in the MZT, or early embryonic development. We isolated this subset by selecting for only those transcripts that exhibit significant changes in expression before and after 12 HPF, and that also have a STEM profile of 31 (peak only at 12 HPF, Figure 1). 42 genes met these criteria. This subset represents some of the earliest genes to be transcribed by the zygotic genome that are also specific to the blastula stage.

Five genes whose homologues are known to interact in other organisms, which function in body plan formation and neuronal development, were among these 42 transcripts. These include an achaete-scute homologue (ID: 106438), also known as NvashB, which functions in proneural patterning in other organisms [18]. In *N. vectensis*, the spatial and temporal expression of this transcript was studied. Expression was first detected via *in situ* hybridization in the blastula at the oral pole, with less staining in the early gastrula, and loss of staining by mid-gastrula [4], these results agree with our findings. In *D. melanogaster* and humans, Achaete-scute is laterally inhibited by Hairy and a Hairy-related protein HES-1, respectively [19,20]. A *N. vectensis* homologue of *hairy* (ID: 242118) also shows increasing expression exclusively in the 7–12 HPF interval. A LIM class homeodomain transcription factor (ID: 246590) known in *D. Melanogaster* as Chip, which interacts with Achaete-scute in proneural prepattern and thorax development [21], is also upregulated only at 12 HPF. Lastly, we identified two *wnt* genes in this subset (ID:115036; ID: 195613), both similar to *wnt8* in other organisms. *Wnt8* is among the earliest zygotically-expressed regulatory factor in the sea urchin, where it is responsible for patterning along the animal-vegetal axis [22,23]. *Wnt8b* expression in humans and mice is restricted to early brain development [24], and in the spider *Achaearanea tepidariorum*, *wnt8* knockdowns affect expression of *hairy*, among others transcripts, and decrease formation of posterior body regions [25]. While multiple *N. vectensis wnts* have been studied with *in situ* hybridization, including one *wnt8*[5] (NCBI ID: AY725205), the transcripts we identify have not been examined. The *wnt8* transcripts we observe at this time point are strongly expressed at 12 HPF, with one (*wnt8* (ID: 115036)) being expressed almost exclusively at this time (corresponding to Wnt8a [5], NCBI ID: AY792510), and another (*wnt8* (ID: 195613)) displaying low level expression before and after 12 HPF.

Wnt8, Hairy, Chip, and Achaete-scute are known to interact in other organisms, and play a role in both body plan patterning and nervous system development. In *N. vectensis* it is possible that they play a role in one or both of these processes. Examining the spatial expression of more of these genes, as well as conducting functional studies, would further shed light on this hypothesis.

#### The onset of gastrulation

We chose to sample gastrula at several hours past the initiation of gastrulation [2], in an attempt to capture early gastrula gene expression. *N. vectensis* gastrulation occurs via invagination [26], and the formation of a blastopore was clearly visible in some embryos.

Of the 2,575 transcripts that increase between 12–24 HPF, 170 have a significant expression peak only at 24 HPF (significantly increasing before and after 24 HPF, and a STEM profile of 28). These genes include a homologue of homeobrain (ID: 165614), a transcription factor that is expressed in brain formation in *D. melanogaster*[27] and the annelid *Capitella teleta*[28]. We also identified a possible frizzled family receptor-10 (ID: 168924), which is also significantly up regulated from 7–12 HPF, and is involved in limb and nervous system development in chicks [29]. These transcripts may be involved in early nervous system and apical organ formation in *N. vectensis*.

## Conclusions

The analyses presented here provide a global perspective on significant changes in gene expression through time during *N. vectensis* development. We identify likely maternal transcripts targeted for degradation, and a subset of transcripts whose proteins may play a role in targeting maternal factors, as well as genes among the first to be transcribed by the *N. vectensis* embryos, which may play a role in neuronal development and/or patterning. We also identified the major wave of zygotic transcription, which occurs after the 128 cell stage between 7–12 HPF. The matrix file (Additional file 2), as well as some suggested approaches for its use (Additional file 3) will allow other investigators to examine temporal changes in transcripts of particular interest, perform additional analyses, and examine time points relevant to processes not directly addressed here. Future applications of RNA-seq to characterize the transcriptional dynamics of *N. vectensis* development will likely benefit by higher temporal resolution. The results presented here will help guide the selection of additional time points so that changes in expression can be pinpointed in time more precisely. In addition, an updated set of transcript predictions will be essential for more detailed analyses. The gene predictions provided by the *N. vectensis* genome project have been an invaluable resource to the community, and enabled many projects (including the one presented here). There are several properties of the gene predictions generated for this project that limit their utility for use in conjunction with new tools, such as RNA-seq, that were not available at the time the genome annotations were produced. In particular, the presence of rRNA in a large number of gene predictions and the absence of multiple known genes limit the analyses that can be done with these sequences. An updated set of gene annotations and transcript predictions, which will surely benefit from the much deeper transcriptome sequencing that is now possible, will be a critical goal for further work with high-throughput tools for the study of *N. vectensis* development and functional genomics.

## Methods

### Spawning and sample collection

Our *N. vectensis* culture was founded with adults from Mark Martindale’s laboratory (University of Hawaii). Animals were kept in 12 parts per thousand seawater (*Nematostella* Medium: NM) at 16°C, fed newly hatched *Artemia* twice weekly, and cleaned once a week. Females were kept in female-only bowls.

A total of two replicates time course were collected in this study. For each time course, a single pool of embryos, spawned from the same parents at the same time, was sampled over the course of 10 days. Spawning was induced by feeding female-only bowls and mixed bowls (with males and females) oyster, followed by a water change and placement on a light box attached to a timer [30]. They were exposed to 8 hours of light, with bowl water temperatures increasing to above 24°C. After light exposure, animals were moved to a dimly lit room, and any eggs spawned overnight were removed. Bowl water was changed to room temperature 0.2 μm filtered NM.

Females began to spawn approximately two hours after light exposure ended. Every 30 minutes newly spawned eggs were moved to small mesh bottom cups in NM from mixed male/female bowls (which contained sperm), kept at 18-23°C. Time of fertilization was considered to be at the time cups were placed in mixed water. Eggs were allowed to sit in water from mixed bowls for one hour. NM from the mixed male/female bowls was changed once over the course of collection. In *N. vectensis*, eggs are secreted in a gelatinous matrix, which must be removed before embryos can be sorted. Embryos were de-jellied by rocking them for 15–30 minutes in 40 ml NM, with 1.6 g cystine and 12 drops 5M NaOH. Embryos were rinsed 3 times with 0.2 μm 18°C filtered NM, divided and placed in 0.2% gelatin coated dishes. A total of six dishes, one for each time point, were prepared. Each dish had approximately 500–1000 embryos. These dishes were kept at 18°C for the duration of development.

We sampled six time points during each replicated time series. The target sampling points were zygote (2 HPF), early blastula (7 HPF), mid-blastula (12 HPF), early gastrula (24 HPF), planula (5 DPF), and young polyp (10 DPF). The exact sampling times had minor variation, and were 2.50, 7.23, 12.23, 23.60, 125.42, and 240.07 HPF for the first replicate spawning, and 2.55, 7.30, 12.48, 23.63, 125.50, 240.13 HPF for the second replicate. Prior to sampling for gene expression, any anomalous or un-cleaved embryos were removed (after 2 HPF), and the remaining embryos were rinsed with 0.2 μm 18°C NM. The fertilization rate was higher than 90% for both replicates. For each time point, approximately 500–1000 embryos were placed in RNAse-free non-stick tubes, excess liquid was drawn off, and they were snap frozen on liquid nitrogen.

### mRNA extraction & HiSeq preparation

Messenger RNA was extracted directly from tissue with Dynabeads from the mRNA Direct Kit (Invitrogen) with only one round of bead hybridization. mRNA was treated with Turbo DNase (Ambion) and concentrated by ethanol precipitation. Re-suspended mRNA samples were quantified with Qubit. Samples were prepared for multiplex sequencing using Illumina TruSeq RNA Sample Prep Kits A and B (part # FC1221002 (kit B), Lot: 5781467) according to manufacturer's instructions, with an RNA fragmentation time of 8 minutes at 94°C.

### Sequencing

All twelve samples were sequenced in a single lane on the Illumina HiSeq 2000 at the Brown Genomics Core Facility. Reads were single-end 50bp, with a separate read to sequence the sample index. Reads were de-multiplexed according to their index sequences with Casava version 1.8 (Illumina). Reads that did not pass the Illumina chastity filter were discarded.

### Reference and mapping

Filtered transcript predictions from the Joint Genome institute (JGI) *N. vectensis* genome project (http://ftp.jgi-psf.org/pub/JGI_data/Nematostella_vectensis/v1.0/annotation/transcripts.Nemve1FilteredModels1.fasta.gz) were used as reference sequences [1]. The original JGI transcriptome file has 27,273 predicted transcripts, with a mean contig length of 1,092 nucleotides. Some of these transcripts contain fragments of ribosomal RNA sequences, which, due to the high expression of ribosomal RNA, could complicate analyses of differential gene expression. We therefore removed transcripts that matched any of the following sequences according to a blastn search with an e-value less than 1e-40: 28S (extracted from the *N. vectensis* genome), 18S (GenBank: AF254382.1) and the mitochondrial genome (including 16S and 12S; GenBank: DQ643835.1). 762 transcripts matched these sequences and were removed, though some gene predictions that include rRNA fragments that did not match with these stringent criteria are still present. The 18S, 28S, and mitochondrial sequences itemized above were then added to the reference, so that the number of reads that mapped to these high-abundance sequences could be quantified. This produced a reference of 26,514 transcripts. The modified reference, including single copies of the ribosomal sequences and the mitochondrial genome, is provided as Additional file 1.

Our sequence reads were mapped to the reference using bowtie 2.2.0 beta3, with the --very-sensitive-local and -a flags. The --very-sensitive-local increases sensitivity at the cost of computational resources, while -a returns all possible mappings for a single read, rather than just the top mapping. A list of full commands used can be found in (Additional file 7). Counts were generated from the bowtie2 map file using an in-house script (Additional file 8). This script does not count any read that maps to more than one reference sequences, and multiple mappings to the same reference sequence are only counted once. This reduces the impact of misassembled transcripts on the derivation of read counts.

### Statistical analyses of expression

Statistical analyses were performed with R version 2.15.2. The R code for the analyses are in Additional files 9 and 10. The matrix with raw read counts, normalized counts, statistical analyses of changes in expression, top blast hit, and other annotations is in Additional file 2. This file includes all reads present in the filtered transcript predictions from the *N. vectensis* genome project, except redundant reads matching our query ribosomal sequences, which were removed (as discussed in “Reference and mapping”). One copy each of 18S, 28S and the mitochondrial genome were manually added after statistical analysis. This matrix can be used to examine genes and time points not presented in the main text.

We tested the significance of differential expression between each pair of adjacent sampling time points, using the R library edgeR version 3.0.4 [31]. Since there were six sampling time points, there were five intervals that were tested. Transcripts without read counts or with very low read counts were filtered out before performing the test. This filtering strategy aimed at keeping transcripts with an average read count of at least 1 count per million (CPM) for replicates of a particular time point (keep <− rowSums(cpm(d) > 1) >= 2). TMM normalization was applied to account for compositional difference between libraries using the function calcNormFactors [32]. Dispersion was estimated using the functions estimateCommonDisp and estimateTagewiseDisp. Testing for differential expression was done using the function exactTest. P-values were corrected for multiple testing using p.adjust and Bonferroni correction. We considered genes with an adjusted p-values below 0.05 as differentially expressed.

We refer to an increase or decrease in read counts between time points for a gene as an increase or decrease in expression of the gene. We acknowledge that this is a simplification of terminology, as numerous biological factors can influence mRNA abundance in cells, including mRNA degradation rate, so that read counts are not a strict measure of expression.

### STEM analysis

Temporal patterns of expression for individual transcripts were categorized and visualized with Short Time-series Expression Miner (STEM) version v1.3.8 [33]. These analyses were performed on normalized count data, averaged between replicates. Normalized data were generated by multiplying each count by a normalization multiplier (generated by dividing 1 million by the multiple of the library size and a normalization factor generated using the EdgeR package (using the calcNormFactors function)). Data were input with the Normalized Data option. The data were fit to 50 possible expression profiles, with the maximum unit change between two time points set to two. The STEM output is in Additional file 6, and the profiles it produced are shown in Additional file 4.

STEM profiles only measure abundance, and do not assess significance. To identify genes with a significant peak of expression at a particular time point, we first used STEM profiles to identify all genes with a pattern of interest, then evaluated the significance of the change in abundance for each transcript, keeping only those transcripts that met our significance criteria.

### GOseq analysis

The reference was compared with blastx to the metazoan sequences in the NCBI nr protein database with an e-value cutoff of 1e-5. Specific GO annotations were then produced with the blast2go command line interface [34] and a local instance of the blast2go database (version b2g_may10). The blast2GO output was modified to fit the gene2GO format used by the R package topGO version 2.10.0 (Additional file 11) [35]. We used functionality of topGO to build acyclic graphs for the three domains, biological process (BP), molecular function (MF) and cellular compartment (CC), based on the blast2go annotations. After building the graphs the complete set of annotations were exported using the function genesInTerm. The resulting file (Additional file 12) in combination with the reference matrix (Additional file 2) allows for the retrieval and subset analysis of transcripts that received a particular GO term.

A gene set enrichment analysis was performed using the R package GOseq version 1.10.1 [36]. Adjusted p-values from the edgeR analysis and a cutoff of 0.05 were used to construct a numeric vector of differentially expressed genes. Annotations in Additional file 12 were loaded as category mappings. Weightings for each gene, depending on its length, were obtained using the probability weighting function nullp and over and underrepresented GO categories were calculated using the Wallenius approximation. P-values were adjusted using p.adjust and the Benjamini and Hochberg method. We considered categories with an adjusted p-value below 0.05 as enriched. The complete list of enriched GO terms for each GOseq analysis are in Additional file 5. The R code for this analysis can be found in Additional file 10.

## Abbreviations

HPF: Hours post fertilization; DPF: Days post fertilization; DGE: Differential gene expression that is significant (adjusted p < 0.05); NM: *Nematostella* medium (12 parts per thousand salinity); GO: Gene ontology; ID: JGI transcript ID; STEM: Short time-series expression miner; MZT: Maternal to zygotic transition.

## Competing interests

The authors declare that they have no competing interests.

## Authors' contributions

CWD, RRH, ST and JS designed the study; RRH collected samples and prepared libraries; RRH, SS, and CWD analyzed data, RRH and CWD wrote the manuscript. All authors read and approved the final manuscript.

## Supplementary Material

Additional file 1 **Reference sequences.** This file is based on the genome project transcript predictions (http://ftp.jgi-psf.org/pub/JGI_data/Nematostella_vectensis/v1.0/annotation/transcripts.Nemve1FilteredModels1.fasta.gz), as described in the methods. This reference contains 26,514 sequences, and was used for mapping. This is a zipped fasta file. Click here for file

Additional file 2 **Data matrix.** Matrix file containing the complete count data, statistics, and annotated results from various analyses (STEM, GO etc.). Detailed information is provided in the comments that precede the file header line. This is a zipped text file. Click here for file

Additional file 3 **Example Analyses.** Example analyses of the matrix and other additional files. These analyses can be used as a starting point for additional examination of the data. This file is a zipped folder that contains a pdf document and the knitr source code that computes the pdf from the data. The last section of the pdf explains how to recompile the document from the source code and data. Click here for file

Additional file 4 **STEM Profiles.** Plots of each profile identified by STEM for our dataset, with associated profile number. A subset of the most highly represented of these plots is shown in Figure 1. The vertical axis is relative transcript abundance, and the horizontal axis is relative developmental time, from the first time point (2 HPF) on the left and the last (10 DPF) on the right. This is an image file. Click here for file

Additional file 5 **Enriched GO terms in different intervals.** The complete list of enriched GO terms from all performed GOseq analyses (adjusted p < 0.05). Additional information is provided in the comments that precede the file header line. This is a text file. Click here for file

Additional file 6 **STEM output.** The “Main Gene Table” output of the STEM program. This is a text file. Click here for file

Additional file 7 **Bowtie2 Commands.** The shell calls for Bowtie2 mapping. This is a text file. Click here for file

Additional file 8 **Python program for converting Bowtie2 output to count data.** This Python program takes in a .sam mapping file generated by bowtie2 and returns the number of reads that map to each transcript. It discards reads that map to multiple sequences, and reads that map multiple times to the same sequence are counted only once. This is a text file. Click here for file

Additional file 9 **Matrix generation code.** The R code and regular expressions used to populate the matrix with count data, normalized and averaged counts, BLAST annotations, STEM profiles, UniProt annotations, JGI numbers, and KEGG annotations. Statistical analyses were performed separately (see Additional file 10). This is a text file. Click here for file

Additional file 10 **R code statistical analysis with edgeR and GOseq.** The R code for performing differential expression tests with edgeR and gene set enrichment analysis with GOseq. This is a text file. Click here for file

Additional file 11 **Blast2GO annotations.** The Blast2GO annotations modified to fit the gene2GO format used by topGO. To generate this file, the reference was used as a query and BLAST against the nr database (e-value cutoff of 1e-5). Transcripts were subsequently annotated with GO terms using the Blast2GO Pipeline. The header row describes the file contents. This is a text file. Click here for file

Additional file 12 **GO-transcript annotations.** This file contains the complete set of GO-transcript annotations as derived from the GO graph that was built by topGO. GO terms were extracted using the topGO function genesInTerm. This file is more inclusive than Additional file 11 (Blast2GO annotations file) as it also contains terms of higher ranks. This is a zipped text file. Click here for file
