# Supplementary material for: Characterization of differential transcript abundance through time during Nematostella vectensis development
Source: BMC Genomics. 2013 Apr 19;14:266. doi: 10.1186/1471-2164-14-266 (PMC3680055; doi:10.1186/1471-2164-14-266)
Supplement: Additional file 3 — Example Analyses. Example analyses of the matrix and other additional files. These analyses can be used as a starting point for additional examination of the data. This file is a zipped folder that contains a pdf document and the knitr source code that computes the pdf from the data. The last section of the pdf explains how to recompile the document from the source code and data. [file 1471-2164-14-266-S3.zip › additional file 3.pdf]

Exploratory Analyses of Additional Data Files

Helm *et al.*

December 27, 2012

Contents

1 Introduction **1**

2 Getting set up **1**

3 Visualizing the data **2**

4 Significance of differential expression **4**

5 STEM profiles **6**

6 Gene Ontology Annotations **6**

7 How this document was made **8**

1 Introduction

This document presents a series of sample analyses of the other additional data files. This pdf has been computed from the data in the Additional files with the source code in Additional file 13, using knitr, R, and TeXShop, as described in the final section of this document.

While some of these analyses are drawn directly from results presented in the paper, most are examples of specific follow-up questions that are likely to be of interest.

2 Getting set up

The first thing we need to do is load the matrix and other data files. Open R and execute the following commands (substitute the relative paths shown here with the paths to the Additional files you have downloaded):

```
m <- read.table("../Additional_file_7_matrix.txt", header = TRUE)
go <- read.table("../Additional_file_10_GO_transcript_annots.txt", header = TRUE)
```

In the matrix, now loaded as m, each transcript is a row and each column has transcript-specific data and analysis results. The following shows the names of the matrix columns:

```
names(m)

#  [1] "transcript"                        "length"
#  [3] "count_rep1_2h"                     "count_rep2_2h"
#  [5] "count_rep1_7h"                     "count_rep2_7h"
#  [7] "count_rep1_12h"                    "count_rep2_12h"
#  [9] "count_rep1_24h"                    "count_rep2_24h"
# [11] "count_rep1_5d"                     "count_rep2_5d"
# [13] "count_rep1_10d"                    "count_rep2_10d"
# [15] "normalized_count_rep1_2h"          "normalized_count_rep2_2h"
# [17] "normalized_count_rep1_7h"          "normalized_count_rep2_7h"
# [19] "normalized_count_rep1_12h"         "normalized_count_rep2_12h"
# [21] "normalized_count_rep1_24h"         "normalized_count_rep2_24h"
# [23] "normalized_count_rep1_5d"          "normalized_count_rep2_5d"
# [25] "normalized_count_rep1_10d"         "normalized_count_rep2_10d"
# [27] "average_normalized_count_2h"       "average_normalized_count_7h"
# [29] "average_normalized_count_12h"      "average_normalized_count_24h"
# [31] "average_normalized_count_5d"       "average_normalized_count_10d"
# [33] "logFC_2h_7h"                       "logCPM_2h_7h"
# [35] "PValue_2h_7h"                      "P_adjust_2h_7h"
# [37] "logFC_7h_12h"                      "logCPM_7h_12h"
# [39] "PValue_7h_12h"                     "P_adjust_7h_12h"
# [41] "logFC_12h_24h"                     "logCPM_12h_24h"
# [43] "PValue_12h_24h"                    "P_adjust_12h_24h"
# [45] "logFC_24h_5d"                      "logCPM_24h_5d"
# [47] "PValue_24h_5d"                     "P_adjust_24h_5d"
# [49] "logFC_5d_10d"                      "logCPM_5d_10d"
# [51] "PValue_5d_10d"                     "P_adjust_5d_10d"
# [53] "blast_hits"                        "cluster_ID"
# [55] "STEM_profile"                      "jgi_id"
# [57] "GO_ID"                             "KEGG_annotation"
# [59] "UniProt_reference_name"
```

There is a more detailed description of each of these columns at the beginning of the matrix file itself, which you can view by opening it directly in a text editor.

3 Visualizing the data

One of the most informative visualizations of differential expression data is to plot the log fold change against the log of the concentration, as in Figure 2B-F in the main text.

Here is a function that draws such a plot for the second interval (7 HPF-12 HPF), with genes that have significant differences in red:

```
plotInterval2 <- function(D) {
    plot(D$logCPM_7h_12h, D$logFC_7h_12h, pch = 16, xlim = c(-1, 13), ylim = c(-13,
```

```
        13), col = rgb(0, 0, 0, 40, maxColorValue = 255), ylab = "log2FC",
        xlab = "log2CPM")
    points(D[D$P_adjust_7h_12h < 0.05, ]$logCPM_7h_12h, D[D$P_adjust_7h_12h <
        0.05, ]$logFC_7h_12h, pch = 16, col = rgb(255, 0, 0, 70, maxColorValue = 255))
    abline(h = c(-1, 1), col = "grey")
}
```

To make a plot of differential expression for the full matrix with this function, you would do the following:

```
plotInterval2(m)
```

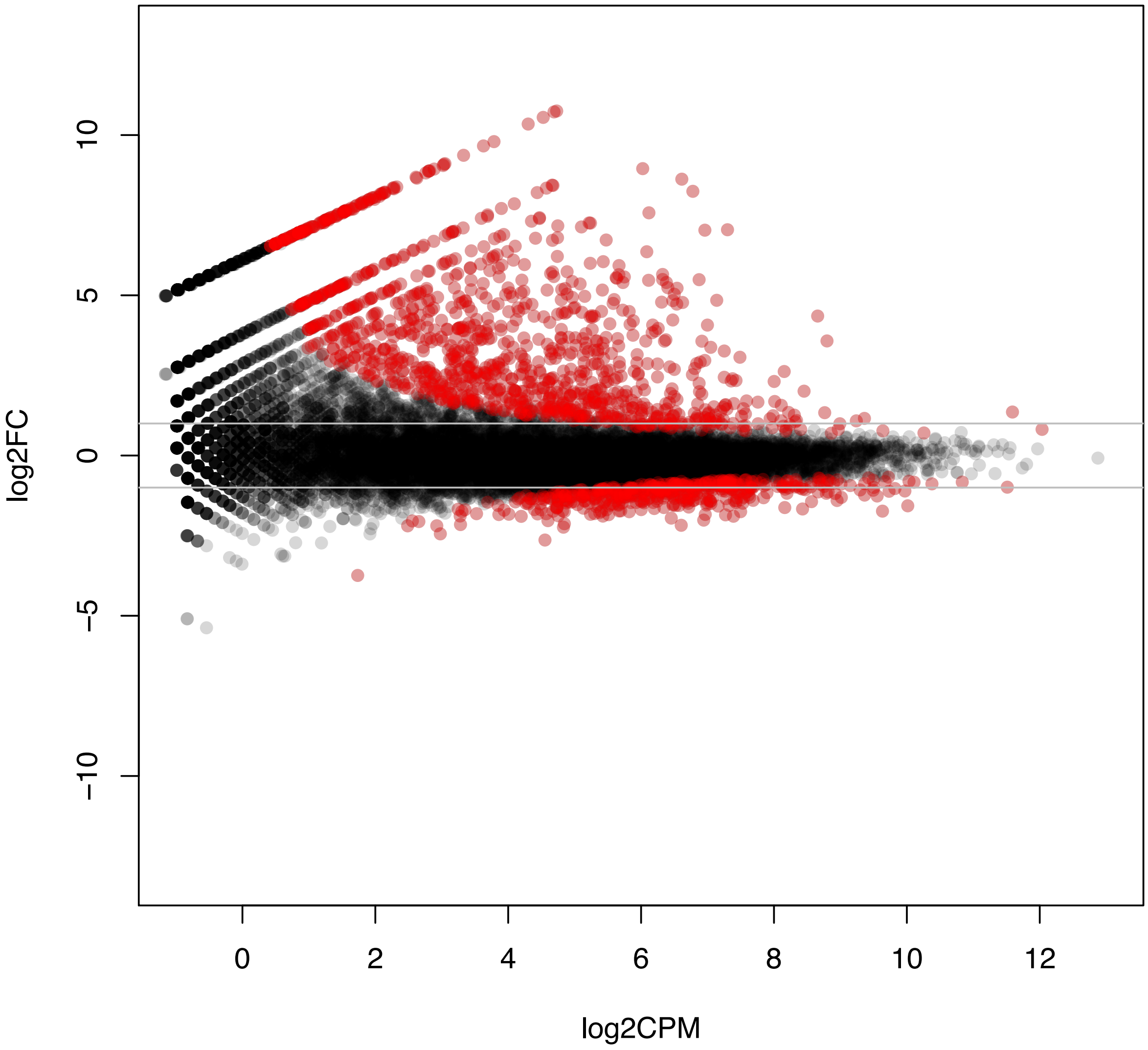

The position along the x axis gives an indication of mean abundance of the transcript. Its position along the y axis gives an indication of the degree of differential expression. Note that genes with no detectable counts at either of the adjacent time points (7 HPF or 12 HPF, in this case) are excluded from the figure. This is because their log fold change is infinite.

You can make similar functions for other intervals by changing the column names in the function above.

4 Significance of differential expression

The significance of differential expression over each interval, as corrected for multiple independent tests, is in the columns that begin with "P_adjust_". For example, the significance of differential expression over the interval from 2 HPF to 7 HPF is in the column labeled "m$P_adjust_2h_7h"

The following isolates the subset of genes with significant differential expression over the first interval and stores this reduced dataset in the new data frame dif2_7:

```
dif2_7 <- subset(m, m$P_adjust_2h_7h < 0.05)
nrow(dif2_7)

# [1] 439
```

The number of rows indicates how many transcripts made it to the subset.

Often you don't just want to know which genes have significant changes, you want to find the genes with significant changes in a particular direction. The direction of change, irrespective of significance, is indicated by the sign of the columns whose names begin with "logFC_". For each transcript, this is the log of expression (ie, normalized counts) at the later interval divided by expression at the earlier interval. This log fold change is positive expression increases over the interval, negative if expression decreases over the interval, and the absolute value indicates the magnitude of the relative change in expression.

To find the genes that not only have a significant change in abundance over the first interval, but are also decreasing, you would use the following:

```
decreasing2_7 <- subset(m, m$P_adjust_2h_7h < 0.05 & m$logFC_2h_7h < 0)
nrow(decreasing2_7)

# [1] 179
```

This is still quite a few genes. You can quickly view the most significant genes in this subset by sorting by P_adjust:

```
# Sort by adjusted P-value
decreasing2_7 <- decreasing2_7[order(decreasing2_7$P_adjust_2h_7h), ]

# Give a synopsis of the top 10 most significant genes
cols <- c(1, 36)  # The indeces of the columns to display
decreasing2_7[1:10, cols]

#                                            transcript P_adjust_2h_7h
# 10519 jgi|Nemve1|203318|fgenesh1_pg.scaffold_42000033      4.653e-21
# 3184                 jgi|Nemve1|126053|e_gw.227.13.1      1.181e-15
# 1801                 jgi|Nemve1|114611|e_gw.127.23.1      1.101e-13
# 21296                   jgi|Nemve1|39952|gw.57.155.1      1.151e-13
# 13428                   jgi|Nemve1|21807|gw.172.60.1      9.874e-13
# 10805 jgi|Nemve1|204929|fgenesh1_pg.scaffold_55000036      5.093e-12
```

```
# 427                      jgi|Nemve1|103667|e_gw.69.225.1      1.769e-11
# 23320                       jgi|Nemve1|71477|gw.33.342.1      8.018e-11
# 24013                      jgi|Nemve1|81132|e_gw.4.455.1      1.059e-10
# 25658                     jgi|Nemve1|93732|e_gw.33.287.1      1.082e-10
```

There are a few ways to get more information about these genes. One would be to download the reference from `ftp://ftp.jgi-psf.org/pub/JGI_data/Nematostella_vectensis/v1.0/annotation/transcripts.Nemve1FilteredModels1.fasta.gz`, search for the corresponding entry with the transcript name, and then take a look at the sequence. There are also names of blast hits right in the matrix, if you would like to get a quick sense of what you have.

5 STEM profiles

Additional file 3 presents the STEM profiles. Each is a particular pattern in the change of transcript abundance through time. STEM profile assignments are included in the main matrix file (Additional file 7) and can be used to filter genes.

As an example, we will consider STEM profile 31. It indicates genes that have peak abundance at the third time point (12 HPF). You could apply the same procedure to other profiles of particular interest.

The following creates a new data frame with only the genes that were assigned to this profile:

```
stem31 <- subset(m, m$STEM_profile == 31)
nrow(stem31)

# [1] 130
```

Not all of the genes that are assigned to this profile will have significant changes. To subset these further to only those that have a significant change before and after the peak, one could use:

```
stem31sig <- subset(stem31, stem31$P_adjust_7h_12h < 0.05 & stem31$P_adjust_12h_24h <
    0.05)
nrow(stem31sig)

# [1] 42
```

6 Gene Ontology Annotations

Since many genes can be assigned to each GO term and each GO term can be assigned to many genes, they aren't stored in the main matrix. Instead, they are in Additional file 10, which was already loaded into the dataframe go.

To find all transcripts in a particular category, you will first need to look up the corresponding GO ID. You can search for these IDs at `http://www.geneontology.org`. In this example, we will explore genes that have a molecular function of "sequence-specific DNA binding transcription factor activity", which have an ID of "GO:0003700".

The first step is to get a vector if the transcript IDs that have an annotation for this category. Next, this vector is used as a filter to create a subset of the original matrix that only has rows for the transcripts that have an annotation in this category.

```
tf <- go[go$GO_ID == "GO:0003700", ]$transcript
mTF <- m[m$transcript %in% tf, ]
```

Now, make a plot of the differential expression of transcription factors over the second interval:

```
plotInterval2(mTF)
```

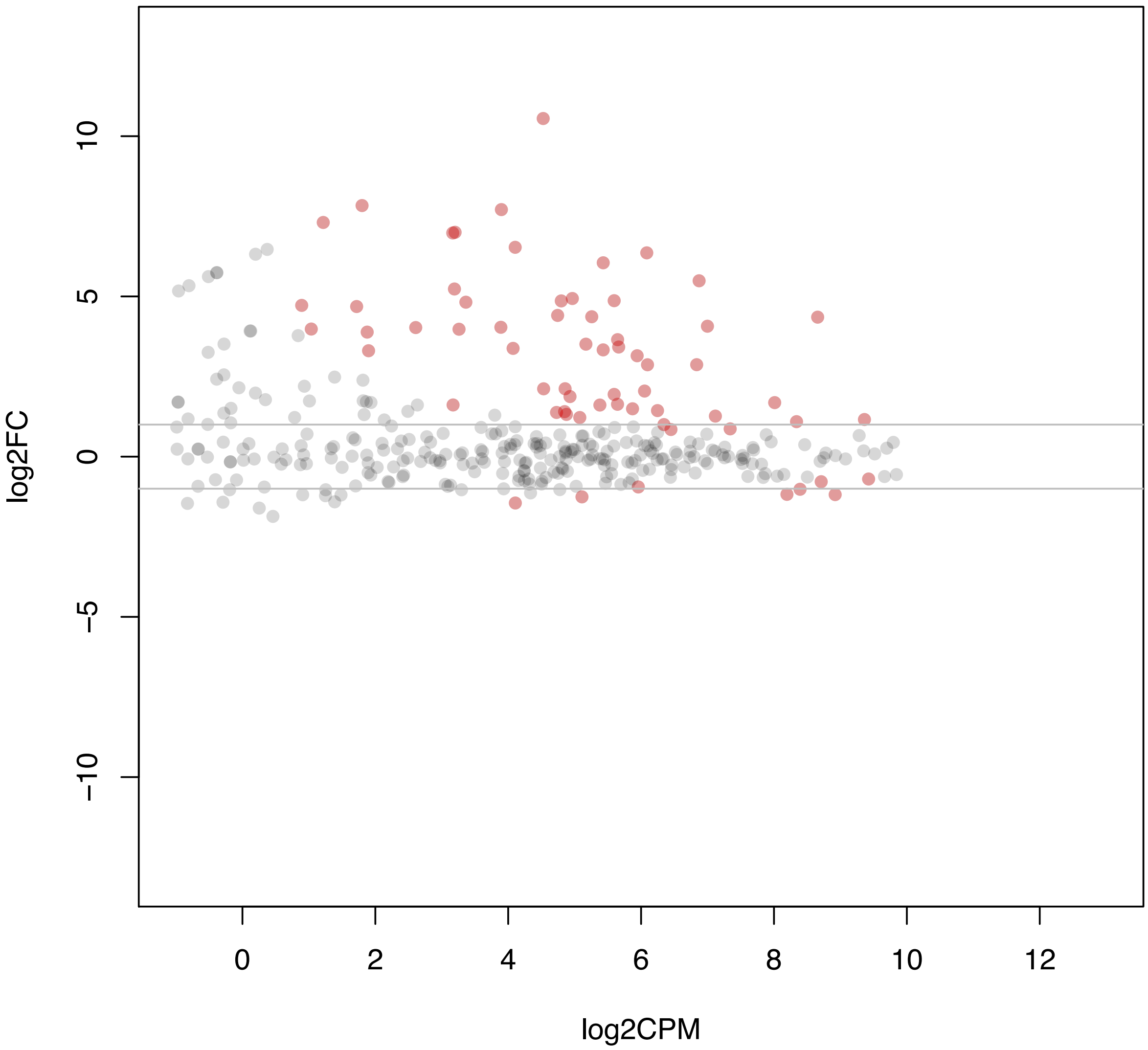

Most of the transcription factors with a significant change in abundance over the second interval (7 HPF - 12 HPF) are increasing in abundance. If you wanted to isolate only the transcription factors with significant increases in abundance over this interval, you could use:

```
passFilter <- (mTF$logFC_7h_12h > 0) & (mTF$P_adjust_7h_12h < 0.05)
passFilter[is.na(passFilter)] <- FALSE
mTF2sig <- mTF[passFilter, ]
nrow(mTF2sig)

# [1] 56
```

7 How this document was made

This document is a computable data report compiled directly from the Additional files. To recreate this file from the data, you will need to install:

- R (http://www.r-project.org). This document was generated with version 2.15.2.
- The R package knitr (http://yihui.name/knitr/), which can be installed from within R. This document was generated with version 0.9.
- pdflatex, which comes with LaTeX distributions (http://www.latex-project.org/ftp.html). This document was generated with version 3.1415926-2.4-1.40.13.

Download the Additional files to a single directory. Unzip Additional file 13, open a terminal window, and cd to the expanded directory. Then launch R, and run the following:

```
library(knitr)
knit("Additional_file_12_example_analyses.Rnw")
quit()
```

This will generate a new tex file. To compile this tex file into a pdf file, run the following at the shell command line:

```
pdflatex Additional_file_12_example_analyses.tex
```

In addition to recreating this document as-is, you can directly edit and add to the analyses in the .Rnw source file. You can also copy the R source code from the .Rnw file.
