# Supplementary figures and images for: Characterization of differential transcript abundance through time during Nematostella vectensis development
Source: BMC Genomics. 2013 Apr 19;14:266. doi: 10.1186/1471-2164-14-266 (PMC3680055; doi:10.1186/1471-2164-14-266)

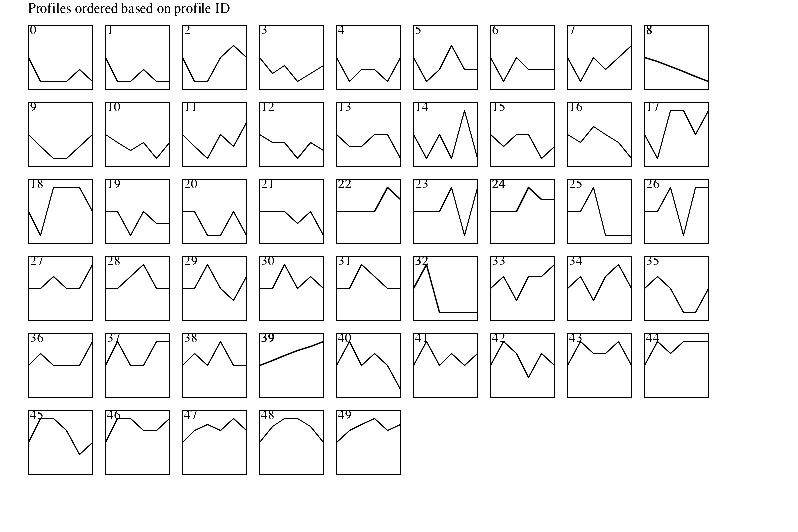

Supplement: Additional file 4 — STEM Profiles. Plots of each profile identified by STEM for our dataset, with associated profile number. A subset of the most highly represented of these plots is shown in Figure 1. The vertical axis is relative transcript abundance, and the horizontal axis is relative developmental time, from the first time point (2 HPF) on the left and the last (10 DPF) on the right. This is an image file. [file 1471-2164-14-266-S4.png]
